# Lingual Varix: Case Series

**DOI:** 10.7759/cureus.116286

**Published:** 2026-09-15

**Authors:** Ramachandra Reddy Gowda Venkatesha, Karthik Rajaram Mohan, Ezhilarasi Arumugam Venkatachalam Sargurunathan, Reethika Rathan, Kumar Appusamy

**Affiliations:** 1 Oral Medicine and Radiology, Vinayaka Mission's Sankarachariyar Dental College, Vinayaka Mission's Research Foundation (DU), Salem, IND; 2 Oral Medicine and Radiology, Vinayaka Mission’s Sankarachariyar Dental College, Vinayaka Mission’s Research Foundation (DU), Salem, IND

**Keywords:** diascopy, phlebothrombosis, sublingual varix, tongue, ventral surface

## Abstract

Varices involving the lingual ranine veins are relatively uncommon, and they clinically appear as red or purple shot-like clusters of vessels on the floor of the mouth as well as on the ventral surfaces of the tongue. Their development may occur as a result of age-related elastolytic degeneration of the elastic tissue within tunica intima and loss of the connective tissue tone. Clinically, lingual varices are asymptomatic and present as a red, purple, or black nodule or bubble that may feel like a piece of buckshot beneath the mucosal surface. This article enlightens us about the case series of lingual varix and its varied clinical appearance.

## Introduction

Varices or varicosities are abnormal, dilated, and tortuous veins [1]. Oral varices can occur in the sublingual region on the floor of the mouth. Usually, such sublingual varices tend to occur over 50 years of age. If such varices are seen prior to 50 years of age, they indicate premature aging [1]. Varices are usually asymptomatic and are accidentally discovered by a dentist during a routine dental checkup [1,2]. Some attributed its pathogenesis to chronic smoking, trauma from ill-fitting dentures, and vitamin c deficiency. Some authors attribute portal hypertension, secondary to liver cirrhosis, as the cause of lingual varices [1,2].

## Case presentation

Case one

A 21-year-old female patient reported to our Department of Oral Medicine for a routine dental checkup. She has no history of tobacco chewing or smoking. Her past dental history revealed she had restoration of her decayed lower right back tooth one year back. Her medical history revealed that she was not hypertensive, diabetic, or asthmatic. Her drug history disclosed no use of oral anticoagulants. A general examination indicated her vital signs were stable. On intraoral clinical examination, multiple discrete dark red-colored buckshot areas measuring about 1 mm x 2 mm were observed on the floor of the mouth (Figure 1).

**Figure 1:**
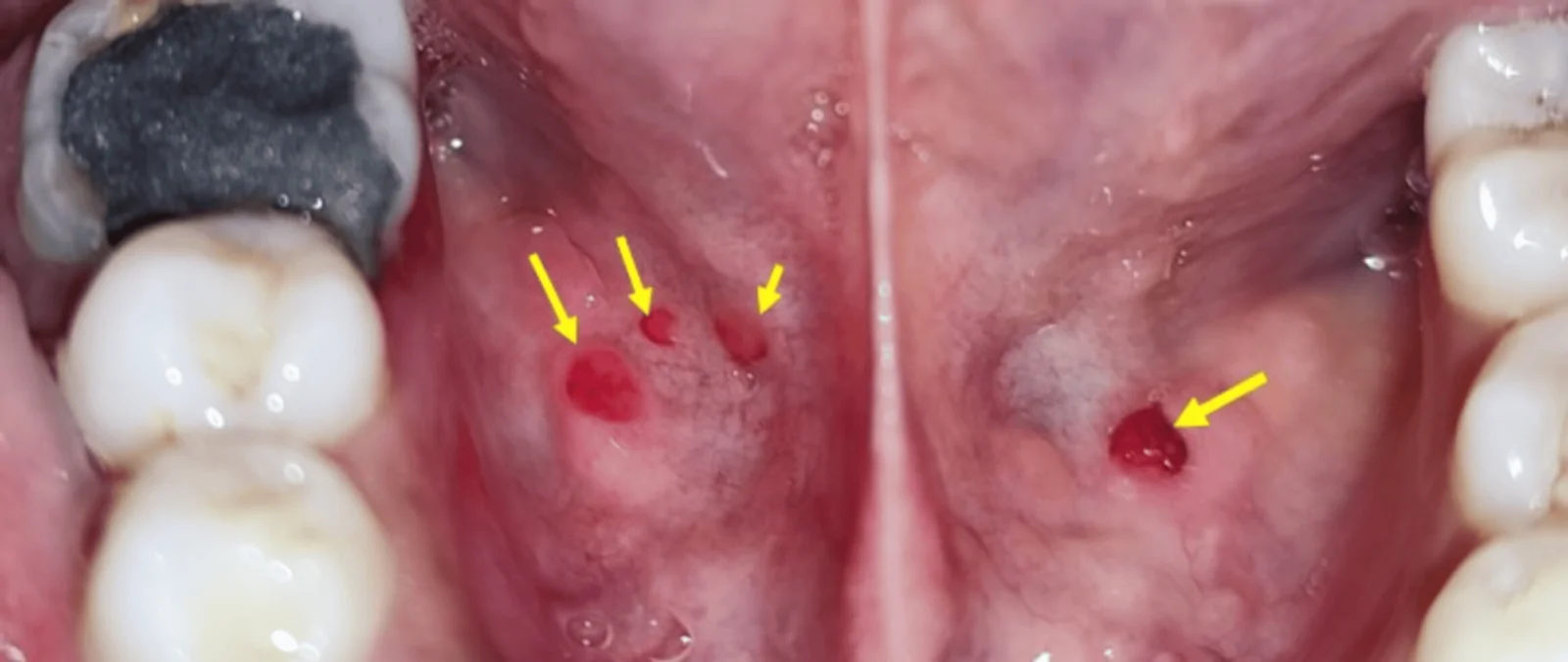
Intraoral clinical examination revealed lingual varicosities appearing as multiple discrete dark red-colored buckshot areas measuring about 1 mm x 2 mm on the floor of the mouth

The patient was completely asymptomatic. No tenderness or bleeding was present on palpation. Her blood coagulation profile was within biological ranges.

Case two

A 27-year-old female patient reported for a routine dental checkup. The patient gives a history of an accidental tongue-biting six months back. Her medical history was non-contributory. Her medical history revealed no intake of oral anticoagulants. Her vitals are stable. Extraoral examination revealed no cervical lymphadenopathy. On intraoral examination, an intraoral swelling measuring approximately 2 mm x 4 mm on the ventral surface of the tongue, which is dome-shaped was observed; the surface of the swelling appeared greyish, shiny, and stretched (Figure 2).

**Figure 2:**
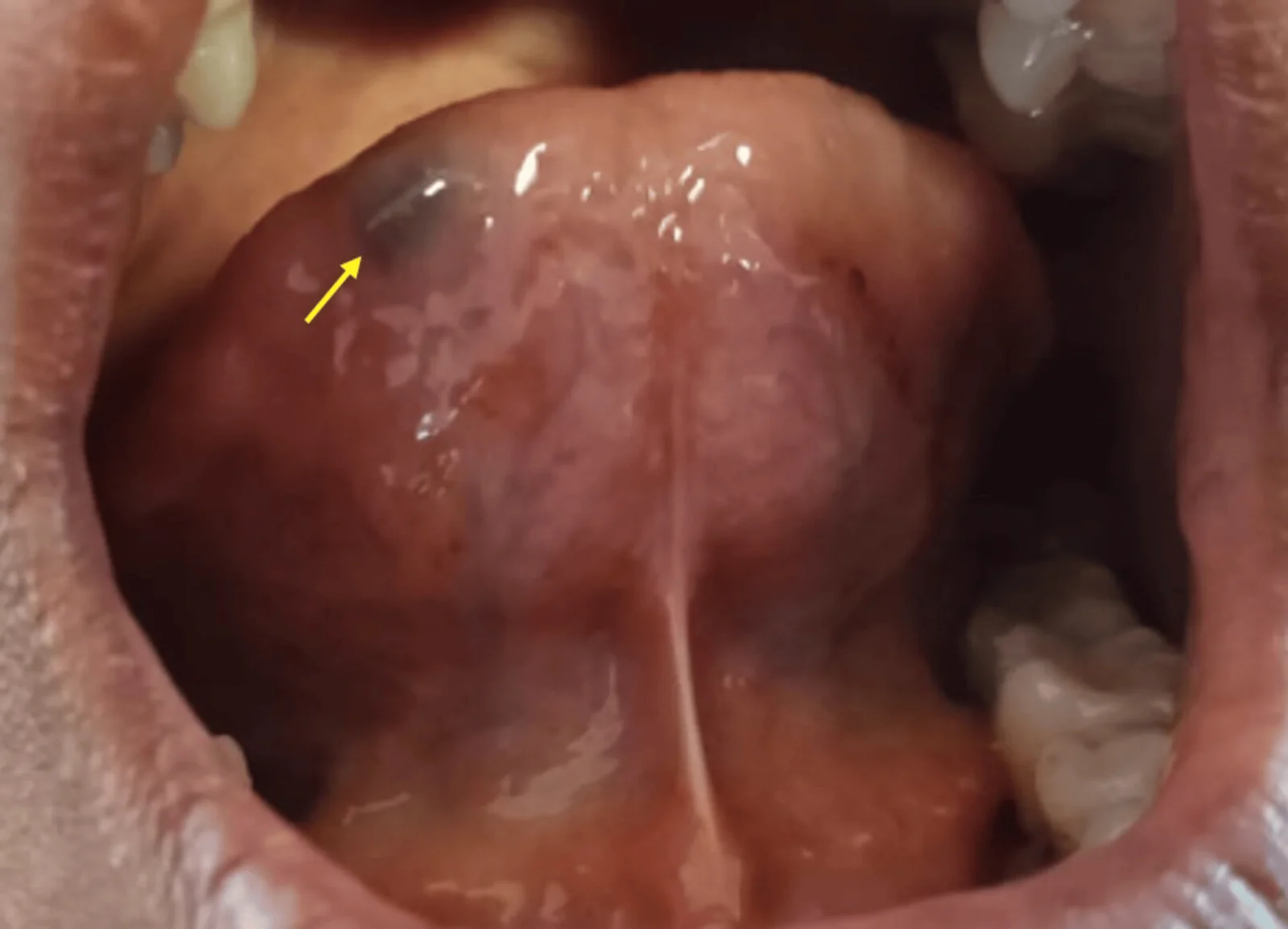
Intraoral clinical examination revealed an intraoral swelling about 2 mm x 4 mm on the right ventral surface of the tongue (yellow arrow) The surface of the swelling appears shiny and stretched.

No associated pain, paresthesia, or difficulty in tongue movements. On palpation, it is non-tender and soft in consistency. A diascopy test did not reveal any blanching (Figure 3).

**Figure 3:**
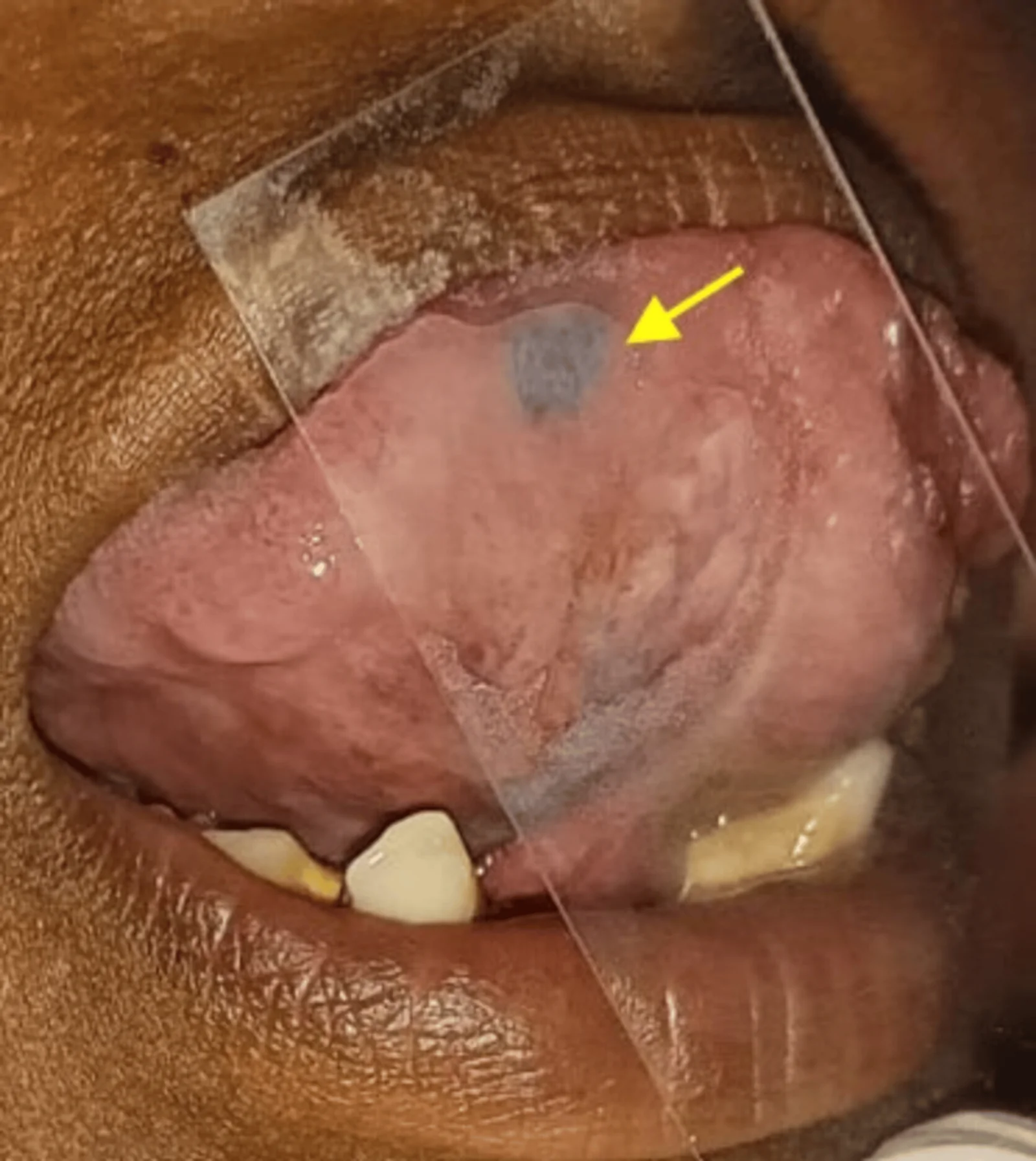
Diascopy test did not reveal blanching (yellow arrow) Diascopy is a chairside procedure done by the application of gentle pressure on the suspected colored lesion on the oral mucosa using a microscopic glass slide.

Her blood coagulation profile was within biological ranges. The differential diagnosis of lingual varix includes mucocele, phlebothrombosis, and oral melanotic macule. Mucoceles, which are superficially located, usually appear dome-shaped with mild bluish tinge due to the presence of mucin. Phlebothrombosis is a blood clot or thrombi formed within the dilated veins. When a varix is filled with thrombi, it does not show positive diascopy tests. Oral melanotic macules appear clinically as flat, non-elevated, dark-brown-colored areas with irregular outlines that can reach a considerable size of 25 mm x 15 mm on the dorsum of the tongue. The clinical significance of the above-mentioned cases is that they highlight the varied clinical appearances of lingual varix, which can be mistaken for other pigmented lesions such as vascular malformations. Such lingual varix do not require any surgical intervention as they are asymptomatic and do not cause any harm.

## Discussion

Burket stated that "related incidences of disorders of the cardiorespiratory system have not been proven" in his patients with varicosities of the lingual veins [1]. Varicosities can help the dentist evaluate his patient because the tongue has long been utilized as a gauge of metabolic activity. Younger age groups with varicosities may be experiencing early physiologic change [1]. The term "Varix" was derived from the proto-indo European word "wers" meaning "highland" or "high," and ancient Greek "herma" meaning "reef," "rock," or hill." It was known by other names such as caviar tongue phlebectasias [1]. A dilated, enlarged tortuous vein, artery, or lymphatic channel is referred to as a varix. A swollen and tortuous vein, artery, or lymph vessel is known as a varicosity. Orally, varicosities are perceived as clusters of red, purple, or shot-like colors [1]. The dentist's and doctor's observations of lingual varicosities have been attributed to senile elastolytic degeneration of the sublingual veins brought on by aging, tissue relaxation, and elevated venous pressure [1]. Other names for it include lingual varicosities, caviar lesions, and sublingual varices. The consensus is that they are acquired and innocuous. Varicosities were not shown to be directly related to any other specific organic illnesses [1]. Oral melanotic macules clinically occur as flat, non-raised brown areas with irregular outlines on the dorsum of the tongue [2].

The prevalence of sublingual varices was 25.6% at onset and 30.6% during follow-up. During the eight-year follow-up period, 80.9% of the 209 initial patients with no or few sublingual varicosities (SLV) maintained their sublingual vascular status, while 19.1% developed SLV [3]. Histologically, the varix is thin-walled, distended, filled with blood, and bordered with squamous epithelium [4].

Ettinger RL and Manderson RD stated in their study that there exists a substantial relationship between sublingual varices and the presence of leg varicosities [5]. A condition known as varicosity denotes an inflated and convoluted vein. Browne L stated in his note on lingual varix that Dr. Allen described the situation of incredibly enlarged varicose veins in a 70-year-old man who had two episodes of influenza and complained of a terrible cough [6].

According to Kleinman HZ, the occurrence rate of lingual varicosities was twice as high in those with cardiac pulmonary illness compared to those without the disease, and the prevalence of lingual varicosities was shown to be 59% (35 out of 59) in individuals with cardiopulmonary disease, compared to 30% (30 out of 100) in patients without cardiac pulmonary disease. Oral vascular lesions can be seen in Fabry's syndrome (angiokeratoma corporis diffusum universale) and Osler's disease (hereditary hemorrhagic telangiectasia). In contrast to the late occurrence of lingual varicosities, the lesions are discovered in both of these disorders at a young age. Furthermore, locale contributes to distinction. On the tongue's tip and dorsum, Osler's angiomas can be discovered, but Fabry's angiomas are more common in areas such as the thigh, groin, buttocks, and umbilicus [7].

Accardo A et al.(2021) in their study of 1008 participants found that SLV is related to age and noted differences in SLV among people with varying degrees of hypertension. Therefore, the presence of varices may indicate underlying systemic hypertension and such patients can be referred to a general physician for the presence of hypertension [8]. Oral thrombi may be overlooked because they have a minimal risk of embolization and mortality. Currently, there is no evidence of embolization caused by oral thrombi. Approximately 18% of oral cases exhibit the characteristic of tongue involvement, as reported by certain authors [9]. Dermoscopy aids in the diagnosis of SLV [10].

The presence of SLV may serve as an indication of prospective risk factors such as advanced age, female gender, and wearing dentures. It can also notify dental clinicians to be aware of underlying systemic illnesses, specifically cardiovascular disease (CVD) [11]. Dentists should refer patients with SLV to hypertensive specialists after examining them. The data also showed a further relationship with people who had compensated hypertension, demonstrating a substantial correlation between the existence of SLV and subjects with resistant hypertension. Identifying a subject with varicose veins would necessitate reassessing him for blood pressure and all cardiovascular risk factors, such as smoking and dyslipidemia. This is relevant because approximately 40% of the population is either unaware of their hypertension or lacks effective pharmacological control for it [11]. Gaining a more comprehensive understanding of the potential risk factors linked to SLV can help dental clinicians identify underlying systemic diseases that need to be managed promptly and appropriately [11]. Sublingual varices typically do not require treatment but rather merely require the reassurance of their harmless nature [12].

## Conclusions

The SLV can be misdiagnosed as vascular lesions, and any attempt at surgical treatment must be avoided when such lesions are encountered. A thorough knowledge of SLVs is essential for oral physicians to diagnose them. The presence of sublingual varices can indicate underlying systemic illnesses such as hypertension, liver cirrhosis, and dyslipidemia. Sublingual varices usually need no treatment but only reassurance regarding their benign nature.
